# Supplementary material for: The Usefulness of the C2HEST Score in Predicting the Clinical Outcomes of COVID-19 in COPD and Non-COPD Cohorts
Source: Microorganisms. 2024 Jun 20;12(6):1238. doi: 10.3390/microorganisms12061238 (PMC11205505; doi:10.3390/microorganisms12061238)
Supplement: Supplementary file 1 [file microorganisms-12-01238-s001.zip › microorganisms-3036494-supplementary.pdf]

Table S1. Summary of the results of the laboratory parameters measured during hospitalization for the COPD and non-COPD cohorts

**Variables** [units]	**No COPD,** N=1976	**COPD,** N=75
Laboratory results measured on admission		
WBC [10*3/uL]	9.11±11.83	9.41±5.14
LYMPH [10*3/uL]	1.50±8.77	1.16±0.60
NEUTR [10*3/uL]	6.68±4.90	7.61±5.49
EOS [10*3/uL]	0.05±0.11	0.06±0.17
HGB [g/dl]	12.93±2.27	12.99±2.62
MCV [fL]	89.56±6.12	91.79±6.82
MCH [pg]	29.96±2.46	30.30±3.04
MCHC [g/dl]	33.45±1.53	32.98±1.75
RET [10*3/uL]	57.95±34.49	65.49±28.38
PLT [10*3/uL]	231.79±108.39	238.68±107.03
PH	7.42±0.08	7.40±0.06
PO_2 [mmHg]	73.36±36.35	70.42±39.72
PCO_2 [mmHg]	37.61±10.41	39.39±6.18
HCO_3^{STD} [mmol/L]	24.62±4.16	23.81±3.37
BE(B) [mmol/l]	1.48±5.11	1.61±4.54
LAC [mmol/L]	2.36±1.52	1.84±0.75
Plasma Osmolarity [mOsm/kgH2O]	304.46±20.19	306.70±28.31
Sodium [mmol/L]	138.10±5.44	137.80±5.57
Potassium [mmol/l]	4.10±0.65	4.31±0.76
Calcium [mg/dl]	9.13±0.64	9.24±0.57
Phosphorus [mg/dl]	4.00±1.72	4.45±2.98
Magnesium [mg/dl]	2.07±0.38	2.10±0.36
Chlorides [mmol/l]	102.99±6.38	101.00±6.80
CRP [mg/l]	77.63±84.31	91.49±92.31
Procalcytonin [ng/ml]	1.15±7.30	1.44±3.54
IL-6 [pg/mL]	58.90±359.06	27.39±21.68
ESR [mm/h]	37.98±32.11	25.83±22.80
D-dimers [µg/ml]	4.61±14.22	4.38±12.14
INR	1.24±0.95	1.56±2.30
APTT [sek]	34.87±15.53	34.18±6.60
Fibrinogen [g/l]	4.79±1.82	5.31±1.91
Ferritin [µg/l]	909.47±1508.80	813.55±1227.23
Saturation of transferrin [%]	23.44±20.42	19.39±10.39
Iron [µg/dl]	51.81±41.01	45.93±24.04
sTfR [mg/l]	1.59±0.89	1.19±0.09
Vitamin B12 [pg/ml]	660.73±919.65	460.63±311.27
Folic acid [ng/ml]	7.64±5.20	5.76±4.46
Glucosum [mg/dl]	141.54±84.33	134.11±57.82
HbA1c [%]	7.56±2.18	6.84±1.66
Urea [mg/dl]	52.33±44.02	67.87±50.60
Creatynine [mg/dl]	1.30±1.29	1.63±1.30
eGFR [ml/min]	75.60±35.00	58.88±29.86

Plasma protein [g/dl]	5.96±0.87	6.14±1.00
Albumin [g/dl]	3.11±0.59	3.04±0.70
Uric Acid [mg/dl]	5.92±2.73	5.96±2.00
Creatynin kinase [IU/l]	365.00±1108.12	353.44±572.41
ASPAT [U/l]	65.52±202.35	64.05±59.33
ALAT [U/L]	53.40±132.82	43.71±47.18
Total bilirubin [mg/dl]	0.84±1.23	0.86±0.67
ALP [U/l]	90.91±98.47	88.11±53.32
GGTP [U/L]	84.95±144.81	83.37±91.74
LDH [U/L]	421.71±421.47	443.94±269.62
BNP [pg/ml]	507.02±1288.54	365.27±468.15
NT-proBNP [pg/ml]	7052.27±14880.73	5695.59±5886.99
Troponin [ng/l]	537.75±4969.45	2940.49±17266.97
Cholesterol LDL [mg/dl]	92.27±47.75	76.32±40.94
Cholesterol HDL [mg/dl]	39.48±15.51	39.19±18.72
Triglicerydes [mg/dl]	161.09±109.63	161.10±119.43
25-OH-Vitamin D [ng/ml]	23.96±16.87	25.15±21.54
TSH [uIU/ml]	1.56±2.46	1.43±1.44
FT 4 [pmol/L]	12.86±3.33	12.92±2.21
FT 3 [pg/ml]	2.16±1.57	1.63±0.57
Cortysol [μg/dl]	13.90±14.95	9.02±5.46

Continuous variables are presented as mean ± standard deviation

Table S2. The Log-rank statistics for matching the C_2HEST risk strata for total mortality in COPD cohort.

	h 1	h 2	h 3	h 4	h 5	h 6	h 7	h 8
m 1		0.072	0.2793	0.8754	0.8122	0.4172	0.1034	NA
m 2			0.0804	0.8297	0.6677	0.1923	0.0146	0.072
m 3				1.4894	0.6733	0.3822	0.0797	0.2793
m 4					0.9184	1.5136	0.7853	0.8754
m 5						3.0896	0.7682	0.8122
m 6							0.4082	0.4172
m 7								0.1034
m 8								

Table S3. The Log-rank statistics for matching the C_2HEST risk strata for in-hospital mortality in COPD cohort.

	h 1	h 2	h 3	h 4	h 5	h 6	h 7	h 8
m 1		0.0188	0.3906	0.1046	1.2155	0.2774	1.47	NA
m 2			0.1777	0.0113	1.5275	0.0774	2.1754	0.0188

m 3				0.326	1.5194	0.1747	2.1643	0.3906
m 4					1.9558	0.0771	2.2523	0.1046
m 5						2.4089	2.4949	1.2155
m 6							2.9147	0.2774
m 7								1.47
m 8								

Table S4a. Components of C_2HEST score and primary endpoints in univariate Cox proportional hazard model in COPD cohort.

All cause death				
	HR	CI min.	CI max.	p-value
Coronary artery disease	0.9025845	0.3989066	2.042230	0.8056682
COPD	NA	NA	NA	NA
Age	0.6884068	0.3556242	1.332598	0.2678866
Thyroid disease	0.5730892	0.2103560	1.561311	0.2762860
hypertension	1.0381483	0.4640126	2.322678	0.9273962
HFrEF	1.9459026	0.8934700	4.238012	0.0936745
Hospitalization				
	HR	CI min.	CI max.	p-value
Coronary artery disease	1.5807480	0.4469150	5.591140	0.4774433
COPD	NA	NA	NA	NA
Age	0.5725881	0.2010735	1.630534	0.2963477
Thyroid disease	0.1985660	0.0240561	1.639019	0.1333144
Hypertension	0.7714469	0.2130673	2.793156	0.6926390
HFrEF	2.1528298	0.6292886	7.364946	0.2217475
Discharge from hospital				
	HR	CI min.	CI max.	p-value
Coronary artery disease	3.1843339	0.8073785	12.559144	0.0980555
COPD	NA	NA	NA	NA
Age	1.9149653	0.8131964	4.509479	0.1370769
Thyroid disease	0.5304663	0.1139565	2.469315	0.4191057
Hypertension	0.5685138	0.2253130	1.434484	0.2317345
HFrEF	0.5062773	0.1396843	1.834972	0.3001902

Table S4b. Components of C_2HEST score and selected secondary endpoints and concomitant diseases in univariate Cox proportional hazard model in COPD cohort.

**Endpoint**	**Component**	**OR**	**CI min.**	**CI max.**	**p-value**

**end of hospitalization - full recovery**	Coronary artery disease	1.495	0.353	6.623	0.5839
	Age	2.470	0.903	6.955	0.0805
	Thyroid disease	0.400	0.049	2.147	0.3223
	hypertension	0.501	0.139	1.759	0.2781
	HFrEF	0.565	0.130	2.177	0.4197
	COPD	NA	NA	NA	NA
**end of hospitalization - deterioration**	Coronary artery disease	0.593	0.085	3.507	0.5732
	Age	0.727	0.177	2.592	0.6335
	Thyroid disease	1.506	0.179	9.624	0.6730
	hypertension	0.859	0.205	4.548	0.8432
	HFrEF	0.833	0.147	3.919	0.8245
	COPD	NA	NA	NA	NA
**end of hospitalization - rehabilitation**	Coronary artery disease	0.405	0.058	2.236	0.3186
	Age	0.580	0.142	2.036	0.4124
	Thyroid disease	10.023	1.685	79.753	0.0162
	hypertension	4.060	0.763	38.507	0.1473
	HFrEF	0.821	0.163	3.512	0.7966
	COPD	NA	NA	NA	NA
**end of hospitalization - death**	Coronary artery disease	1.681	0.383	7.343	0.4845
	Age	0.605	0.167	1.958	0.4168
	Thyroid disease	0.153	0.007	1.121	0.1119
	hypertension	0.929	0.223	4.799	0.9231
	HFrEF	2.528	0.615	10.437	0.1916
	COPD	NA	NA	NA	NA
**all-cause shock**	Coronary artery disease	0.426	0.018	4.546	0.5156
	Age	0.577	0.076	3.020	0.5385
	Thyroid disease	NA	NA	NA	NA
	hypertension	0.465	0.080	3.690	0.4110
	HFrEF	1.284	0.127	8.875	0.8106
	COPD	NA	NA	NA	NA
**hypovolemic shock**	Coronary artery disease	NA	NA	NA	NA
	Age	2.095	0.073	64.985	0.6281
	Thyroid disease	NA	NA	NA	NA
	hypertension	0.192	0.006	5.561	0.2806
	HFrEF	NA	NA	NA	NA
	COPD	NA	NA	NA	NA
**cardiogenic shock**	Coronary artery disease	NA	NA	NA	NA
	Age	3.126	0.449	27.423	0.2525
	Thyroid disease	NA	NA	NA	NA

	hypertension	0.261	0.033	2.364	0.1958
	HFrEF	1.536	0.065	16.056	0.7373
	COPD	NA	NA	NA	NA
**septic shock**	Coronary artery disease	0.509	0.019	6.478	0.6232
	Age	0.549	0.026	5.017	0.6202
	Thyroid disease	NA	NA	NA	NA
	hypertension	NA	NA	NA	NA
	HFrEF	2.597	0.214	29.793	0.4263
	COPD	NA	NA	NA	NA
**pulmonary embolism**	Coronary artery disease	0.374	0.012	6.085	0.5060
	Age	1.934	0.205	20.634	0.5474
	Thyroid disease	NA	NA	NA	NA
	hypertension	NA	NA	NA	NA
	HFrEF	3.403	0.229	47.850	0.3428
	COPD	NA	NA	NA	NA
**deep vein thrombosis**	Coronary artery disease	204423273.454	0.000	Inf	0.9991
	Age	404724379.839	0.000	Inf	0.9985
	Thyroid disease	0.257	0.000	Inf	1.0000
	hypertension	316849576.654	0.000	Inf	0.9990
	HFrEF	4.320	0.000	Inf	0.9999
	COPD	NA	NA	NA	NA
**venous thromboembolic disease**	Coronary artery disease	0.374	0.012	6.085	0.5060
	Age	1.934	0.205	20.634	0.5474
	Thyroid disease	NA	NA	NA	NA
	hypertension	NA	NA	NA	NA
	HFrEF	3.403	0.229	47.850	0.3428
	COPD	NA	NA	NA	NA
**myocardial injury**	Coronary artery disease	5.181	1.132	29.860	0.0434
	Age	0.766	0.203	2.698	0.6823
	Thyroid disease	1.457	0.252	9.119	0.6724
	hypertension	0.384	0.069	1.964	0.2505
	HFrEF	0.580	0.109	2.430	0.4795
	COPD	NA	NA	NA	NA
**acute heart failure**	Coronary artery disease	NA	NA	NA	NA
	Age	29542281551925768.0	Inf	Inf	0.9958
	Thyroid disease	1.226	0.000	Inf	1.0000

	hypertension	285654899 70618880.0	0.000	Inf	0.9978
	HFrEF	NA	NA	NA	NA
	COPD	NA	NA	NA	NA
**stroke/TIA**	Coronary artery disease	NA	0.000	NA	NA
	Age	NA	0.000	NA	NA
	Thyroid disease	NA	NA	NA	NA
	hypertension	3.238	0.000	Inf	1.0000
	HFrEF	130565762 9.651	0.000	Inf	0.9985
	COPD	NA	NA	NA	NA
**complete respiratory failure**	Coronary artery disease	1.170	0.104	13.279	0.8944
	Age	0.665	0.064	5.896	0.7142
	Thyroid disease	NA	NA	NA	NA
	hypertension	NA	NA	NA	NA
	HFrEF	1.170	0.104	13.279	0.8944
	COPD	NA	NA	NA	NA
**SIRS**	Coronary artery disease	2.911	0.381	25.030	0.3112
	Age	1.633	0.370	7.244	0.5071
	Thyroid disease	0.414	0.018	3.518	0.4767
	hypertension	0.250	0.053	1.206	0.0742
	HFrEF	0.554	0.057	3.943	0.5804
	COPD	NA	NA	NA	NA
**sepsis**	Coronary artery disease	0.538	0.010	22.065	0.7255
	Age	1.293	0.032	67.289	0.8841
	Thyroid disease	NA	NA	NA	NA
	hypertension	NA	NA	NA	NA
	HFrEF	NA	NA	NA	NA
	COPD	NA	NA	NA	NA
**acute liver dysfunction**	Coronary artery disease	NA	NA	NA	NA
	Age	NA	0.000	NA	NA
	Thyroid disease	NA	0.000	NA	NA
	hypertension	NA	NA	NA	NA
	HFrEF	NA	NA	NA	NA
	COPD	NA	NA	NA	NA
**MODS**	Coronary artery disease	NA	NA	NA	NA
	Age	5.000	0.126	271.562	0.3683
	Thyroid disease	NA	NA	NA	NA
	hypertension	NA	0.000	NA	NA
	HFrEF	NA	0.000	NA	NA

all bleedings	COPD	NA	NA	NA	NA
	Coronary artery disease	0.691	0.052	10.552	0.7733
	Age	1.752	0.160	19.946	0.6270
	Thyroid disease	1.762	0.064	29.208	0.6930
	hypertension	1.099	0.086	36.712	0.9475
	HFrEF	5.683	0.351	149.500	0.2173
	COPD	NA	NA	NA	NA
**respiratory tract bleeding**	Coronary artery disease	204423273.454	0.000	Inf	0.9991
	Age	404724379.839	0.000	Inf	0.9985
	Thyroid disease	0.257	0.000	Inf	1.0000
	hypertension	316849576.654	0.000	Inf	0.9990
	HFrEF	4.320	0.000	Inf	0.9999
	COPD	NA	NA	NA	NA
**upper-GI-tract bleeding**	Coronary artery disease	0.326	0.007	10.490	0.4928
	Age	0.884	0.009	54.713	0.9524
	Thyroid disease	3.169	0.064	212.134	0.5372
	hypertension	0.334	0.002	50.201	0.6288
	HFrEF	NA	NA	NA	NA
	COPD	NA	NA	NA	NA
**urinary tract bleeding**	Coronary artery disease	NA	NA	NA	NA
	Age	NA	NA	NA	NA
	Thyroid disease	NA	NA	NA	NA
	hypertension	NA	NA	NA	NA
	HFrEF	NA	NA	NA	NA
	COPD	NA	NA	NA	NA
**pneumonia**	Coronary artery disease	0.870	0.170	4.381	0.8637
	Age	0.877	0.292	2.691	0.8145
	Thyroid disease	0.707	0.133	4.225	0.6857
	hypertension	2.003	0.551	7.078	0.2786
	HFrEF	3.131	0.731	17.116	0.1484
	COPD	NA	NA	NA	NA
**new cognitive signs and symptoms**	Coronary artery disease	0.808	0.060	11.723	0.8675
	Age	0.411	0.016	4.397	0.5018
	Thyroid disease	1.136	0.041	16.252	0.9281
	hypertension	0.569	0.038	15.721	0.6867
	HFrEF	6.375	0.447	170.037	0.1829
	COPD	NA	NA	NA	NA

Table S5a. Impact of replacement of the general definition of "thyroid disease" with the more precise term "hypothyroidism" on C_2HEST score sensitivity to primary endpoints in COPD cohort.

**All cause death**				
	HR	CI min.	CI max	p-value
Overall	1.0628792	0.8902696	1.268955	0.5000235
low vs medium	0.4599245	0.0595455	3.552417	0.4564873
low vs high	0.5527079	0.0745420	4.098172	0.5618802
**Death during hospitalization**				
	HR	CI min	CI max.	p-value
Overall	1.1872366	0.8952667	1.574426	0.2333597
low vs medium	0.6745365	0.2397008	1.898197	0.4557500
low vs high	NA	NA	NA	NA
**Discharge from hospital**				
	HR	CI min.	CI max.	p-value
Overall	1.153273	0.9004443	1.477092	0.2587206
low vs medium	NA	NA	NA	NA
low vs high	NA	NA	NA	NA

Table S5b. Impact of replacement of the general definition of "thyroid disease" with the more precise term "hypothyroidism" on C_2HEST score sensitivity to selected secondary endpoints and concomitant diseases in COPD cohort.

Endpoint	Comparison	OR	CI min.	CI max.	p-value
**end of hospitalization - full recovery**	overall	1.082	0.820	1.432	0.5776
	low vs medium	NA	NA	NA	NA
	low vs high	NA	NA	NA	NA
**end of hospitalization - deterioration**	overall	0.825	0.557	1.179	0.3074
	low vs medium	NA	NA	NA	NA
	low vs high	NA	NA	NA	NA
**end of hospitalization - rehabilitation**	overall	0.830	0.585	1.146	0.2732
	low vs medium	NA	NA	NA	NA
	low vs high	NA	NA	NA	NA
**end of hospitalization - death**	overall	1.241	0.910	1.717	0.1767
	low vs medium	NA	NA	NA	NA
	low vs high	NA	NA	NA	NA
**all-cause shock**	overall	0.812	0.479	1.292	0.4013
	low vs medium	NA	NA	NA	NA
	low vs high	NA	NA	NA	NA
**hypovolemic shock**	overall	0.662	0.203	1.572	0.4031
	low vs medium	NA	NA	NA	NA
	low vs high	NA	NA	NA	NA
**cardiogenic shock**	overall	0.886	0.487	1.506	0.6653
	low vs medium	NA	NA	NA	NA
	low vs high	NA	NA	NA	NA
**septic shock**	overall	1.085	0.587	1.952	0.7822

	low vs medium	NA	NA	NA	NA
	low vs high	NA	NA	NA	NA
**pulmonary embolism**	overall	1.292	0.717	2.376	0.3859
	low vs medium	NA	NA	NA	NA
	low vs high	NA	NA	NA	NA
**deep vein thrombosis**	overall	NA	NA	NA	NA
	low vs medium	NA	NA	NA	NA
	low vs high	NA	NA	NA	NA
**venous thromboembolic disease**	overall	1.292	0.717	2.376	0.3859
	low vs medium	NA	NA	NA	NA
	low vs high	NA	NA	NA	NA
**myocardial injury**	overall	1.066	0.785	1.455	0.6824
	low vs medium	NA	NA	NA	NA
	low vs high	NA	NA	NA	NA
**acute heart failure**	overall	2.849	1.400	7.900	0.0128
	low vs medium	NA	NA	NA	NA
	low vs high	NA	NA	NA	NA
**stroke/TIA**	overall	1.176	0.329	4.067	0.7782
	low vs medium	NA	NA	NA	NA
	low vs high	NA	NA	NA	NA
**complete respiratory failure**	overall	0.984	0.556	1.750	0.9551
	medium vs high	0.286	0.013	2.536	0.3092
**SIRS**	overall	1.073	0.721	1.583	0.7206
	low vs medium	NA	NA	NA	NA
	low vs high	NA	NA	NA	NA
**sepsis**	overall	1.605	0.665	5.420	0.3386
	medium vs high	NA	NA	NA	NA
**acute kidney injury**	overall	1.022	0.721	1.435	0.9006
	low vs medium	NA	NA	NA	NA
	low vs high	NA	NA	NA	NA
**acute liver dysfunction**	overall	1.677	0.525	7.820	0.3880
	low vs medium	NA	NA	NA	NA
	low vs high	NA	NA	NA	NA
**MODS**	overall	0.498	0.119	1.293	0.2286
	low vs medium	NA	NA	NA	NA
	low vs high	NA	NA	NA	NA
**all bleedings**	overall	1.412	0.787	2.648	0.2495
	low vs medium	NA	NA	NA	NA
	low vs high	NA	NA	NA	NA
**respiratory tract bleeding**	overall	NA	NA	NA	NA
	low vs medium	NA	NA	NA	NA
	low vs high	NA	NA	NA	NA
**upper-GI-tract bleeding**	overall	1.395	0.610	3.420	0.4207
	low vs medium	NA	NA	NA	NA
	low vs high	NA	NA	NA	NA
**urinary tract bleeding**	overall	0.506	0.061	1.828	0.3953
	low vs medium	NA	NA	NA	NA
	low vs high	NA	NA	NA	NA
**pneumonia**	overall	1.268	0.935	1.770	0.1407

	low vs medium	NA	NA	NA	NA
	low vs high	NA	NA	NA	NA
**new cognitive signs and symptoms**	overall	1.185	0.651	2.148	0.5656
	low vs medium	NA	NA	NA	NA
	low vs high	NA	NA	NA	NA

Table S6a. Impact of changing the cut-off point for age to “>65 years” instead “>75 years” on C_2HEST score sensitivity to primary endpoints in COPD cohort.

**All cause death**				
	HR	CI min.	CI max	p-value
Overall	1.0303799	0.8514563	1.246902	0.7584390
low vs medium	1.1086712	0.1362065	9.024181	0.9231772
low vs high	0.8221163	0.1124195	6.012080	0.8469970
**Death during hospitalization**				
	HR	CI min	CI max.	p-value
Overall	1.1052098	0.8066317	1.514308	0.5335596
low vs medium	0.2102418	0.0178167	2.480907	0.2155623
low vs high	0.2033082	0.0255615	1.617051	0.1321428
**Discharge from hospital**				
	HR	CI min.	CI max.	p-value
Overall	1.0147306	0.7588790	1.3568411	0.9214177
low vs medium	0.0145088	0.0007073	0.2976361	0.0060294
low vs high	0.0653177	0.0066470	0.6418551	0.0192699

Table S6b. Impact of changing the cut-off point of age to “>65 years” instead “>75 years” on C_2HEST score sensitivity to selected secondary endpoints and concomitant diseases in COPD cohort.

**Endpoint**	**Comparison**	**OR**	**CI min.**	**CI max.**	**p-value**
end of hospitalization - full recovery	Overall	0.931	0.693	1.240	0.6248
	low vs medium	0.100	0.002	3.791	0.1909
	low vs high	0.676	0.026	17.619	0.7851
end of hospitalization - deterioration	overall	0.972	0.669	1.398	0.8766
	low vs medium	NA	NA	NA	NA
	low vs high	NA	NA	NA	NA
end of hospitalization - rehabilitation	overall	1.012	0.726	1.408	0.9414
	low vs medium	NA	NA	NA	NA
	low vs high	NA	NA	NA	NA
end of hospitalization - death	overall	1.106	0.801	1.536	0.5389

	low vs medium	0.222	0.006	7.236	0.3520
	low vs high	0.319	0.012	8.399	0.4293
all-cause shock	overall	0.701	0.402	1.143	0.1743
	low vs medium	0.222	0.006	7.236	0.3520
	low vs high	0.069	0.002	1.955	0.0757
hypovolemic shock	overall	0.616	0.214	1.492	0.3140
	low vs medium	NA	NA	NA	NA
	low vs high	NA	NA	NA	NA
cardiogenic shock	overall	0.628	0.319	1.123	0.1401
	low vs medium	0.100	0.002	3.791	0.1909
	low vs high	0.051	0.002	1.489	0.0520
septic shock	overall	1.027	0.547	1.913	0.9305
	low vs medium	NA	NA	NA	NA
	low vs high	NA	NA	NA	NA
pulmonary embolism	overall	1.247	0.674	2.399	0.4814
	low vs medium	NA	NA	NA	NA
	low vs high	NA	NA	NA	NA
deep vein thrombosis	overall	3.098	0.740	62.353	0.2540
	low vs medium	NA	NA	NA	NA
	low vs high	NA	NA	NA	NA
venous thromboembolic disease	overall	1.247	0.674	2.399	0.4814
	low vs medium	NA	NA	NA	NA
	low vs high	NA	NA	NA	NA
myocardial injury	overall	1.194	0.867	1.675	0.2857
	low vs medium	NA	NA	NA	NA
	low vs high	NA	NA	NA	NA
acute heart failure	overall	1.715	0.910	3.753	0.1189
	low vs medium	1.000	0.000	Inf	1.0000
	low vs high	NA	NA	NA	NA
stroke/TIA	overall	1.664	0.487	9.706	0.4427
	low vs medium	1.000	0.000	Inf	1.0000
	low vs high	NA	NA	NA	NA

complete respiratory failure	overall	0.923	0.440	1.931	0.8259
	medium vs high	NA	NA	NA	NA
SIRS	overall	0.926	0.607	1.388	0.7114
	low vs medium	0.222	0.006	7.236	0.3520
	low vs high	0.127	0.005	3.455	0.1608
sepsis	overall	1.077	0.317	3.386	0.8912
acute kidney injury	overall	1.234	0.867	1.787	0.2489
	low vs medium	NA	NA	NA	NA
	low vs high	NA	NA	NA	NA
acute liver dysfunction	overall	2.985	0.742	57.902	0.2544
	low vs medium	1.000	0.000	Inf	1.0000
	low vs high	NA	NA	NA	NA
MODS	overall	0.234	0.033	0.746	0.0444
	low vs medium	0.100	0.002	3.791	0.1909
	low vs high	NA	NA	NA	NA
all bleedings	overall	1.378	0.745	2.730	0.3158
	low vs medium	1.000	0.000	Inf	1.0000
	low vs high	NA	NA	NA	NA
respiratory tract bleeding	overall	3.098	0.740	62.353	0.2540
	low vs medium	NA	NA	NA	NA
	low vs high	NA	NA	NA	NA
upper-GI-tract bleeding	overall	1.364	0.575	3.688	0.4835
	low vs medium	1.000	0.000	Inf	1.0000
	low vs high	NA	NA	NA	NA
urinary tract bleeding	overall	0.769	0.187	2.652	0.6801
	low vs medium	1.000	0.000	Inf	1.0000
	low vs high	NA	NA	NA	NA
pneumonia	overall	1.230	0.902	1.719	0.2020
	low vs medium	NA	NA	NA	NA
	low vs high	NA	NA	NA	NA
new cognitive signs and symptoms	overall	1.378	0.745	2.730	0.3158
